# Brief Electrical Stimulation Triggers an Effective Regeneration of Leech CNS

**DOI:** 10.1523/ENEURO.0030-19.2020

**Published:** 2020-06-23

**Authors:** Sharon Cohen, Alon Richter-Levin, Orit Shefi

**Affiliations:** 1 Faculty of Engineering, Bar-Ilan University, Ramat Gan 5290002, Israel; 2 Bar-Ilan Institute of Nanotechnology and Advanced Materials, Bar-Ilan University, Ramat Gan 5290002, Israel; 3 Gonda Multidisciplinary Brain Research Center, Bar-Ilan University, Ramat Gan 5290002, Israel

**Keywords:** CNS, electrical stimulation, glial cells, medicinal leech, regeneration, single cell

## Abstract

The search for therapeutic strategies to promote neuronal regeneration following injuries toward functional recovery is of great importance. Brief low-frequency electrical stimulation (ES) has been reported as a useful method to improve neuronal regeneration in different animal models; however, the effect of ES on single neuron behavior has not been shown. Here, we study the effect of brief ES on neuronal regeneration of the CNS of adult medicinal leeches. Studying the regeneration of selected sets of identified neurons allow us to quantify the ES effect per cell type at the single-cell level. Chains of the CNS that were subjected to cut injury were observed for 3 d, and the spontaneous regeneration was compared with the electrically stimulated injured chains. We show that the ES improves the efficiency of regeneration of Retzius cells, as larger masses of the total branching tree traverse the injury site with better directed growth with no effect on the average branching tree length. No antero-posterior polarity was found along regeneration within the leech CNS. Moreover, the microglial cell distribution was examined revealing more microglial cells in proximity to the stimulation site compared with non-stimulated. Our results lay a foundation for future ES-based neuroregenerative therapies.

## Visual Abstract

**Figure:**
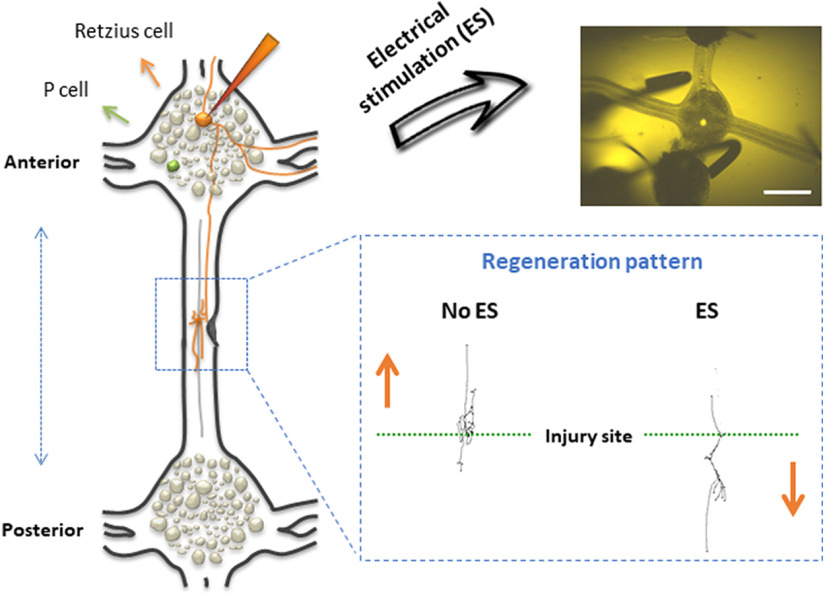

## Significance Statement

Recent studies have demonstrated that brief electrical stimulation (ES) can improve neuronal regeneration. However, the effect of ES on single neuron behavior has not been shown. In the current study, we use a relatively simple nervous system, the adult medicinal leech, label identify neurons, and study the effects of ES on their regeneration. We show that different neurons response differently to the same ES paradigm. Following brief ES (20 Hz, 30 min), more neuronal branches of the Retzius cells traverse the injury site with better directed growth. In addition, more microglial cells were detected in proximity to the stimulation site compared with the non-stimulated nervous systems. We conclude that ES triggers efficient neuronal regeneration and this effect might be mediated through differential microglial distribution.

## Introduction

The central nervous system (CNS) neurons of adult mammals fail to regenerate their lost axons following an injury. In contrast, the peripheral nervous system (PNS) and nervous systems of lower organisms have an intrinsic ability to undergo substantial regeneration (Ferretti et al., 2003; Becker and Becker, 2008; Huebner and Strittmatter, 2009; Mladinic et al., 2009; Ferreira et al., 2012). The unique ability of neuronal tissue to regenerate in some but not other species and what leads nerve regeneration toward functional recovery are longstanding questions that have not yet been solved. Neuronal regeneration has been intensively studied and many efforts have been put into developing methods to increase its effectiveness (Rodríguez et al., 2000; Siemionow and Brzezicki, 2009; Giger et al., 2010; Ferguson and Son, 2011; Baldwin and Giger, 2015). Some of the findings have demonstrated that directing the axonal outgrowth toward appropriate targets by means of physical support, can significantly improve the outcome (Kim et al., 2008; Ferrari et al., 2011; Baranes et al., 2012a,b, 2016; Alon et al., 2014, 2015; Antman-Passig and Shefi, 2016; Marcus et al., 2017). It was also shown that directionality can be achieved by chemical guidance (Dodd and Jessell, 1988; Miller et al., 2002; Rosoff et al., 2004; Kennedy et al., 2006; von Philipsborn et al., 2006; Li et al., 2008; Giger et al., 2010; Lee et al., 2014; Carballo-Molina et al., 2016).

Natural bioelectricity has an important role in many fundamental cellular processes in all cell types (Hoff, 1936; Geddes and Hoff, 1971; Piccolino, 1997; McCaig et al., 2005, 2009; Fixler et al., 2012; Tasset et al., 2012; Podda et al., 2014; Adler et al., 2016; McLean and Verge, 2016). In neurons, ion transporters generate voltage gradients and fluxes that lead to fast dynamic voltage changes, e.g., action potentials, or steady and long-lasting voltage. Upon injury, these natural electrical signals change (Song et al., 2004). Stimulating the electrical activity of the nervous tissue was found to affect neuronal growth (Patel and Poo, 1982; Ming et al., 2001; Wood and Willits, 2006; Ou et al., 2012) and has been suggested as a stimulating mechanism for neuronal repair (Hoffman, 1952; Borgens et al., 1981, 1990, 1999; Nix and Hopf, 1983; Pockett and Gavin, 1985; Borgens, 1999; Song et al., 2004; English et al., 2007; Messerli and Graham, 2011). Recent studies have demonstrated a regenerative effect even when applying brief external electrical stimulation (ES). For example, Al-Majed and colleagues have applied low frequency ES (20 Hz) for time periods ranging from 1 h to two weeks and showed that the ES dramatically accelerated the axonal regrowth of motor neurons and that they were better directed into the appropriate pathways. They have shown that short- and long-term stimulation were equally effective (Al-Majed et al., 2000a). Subsequently, their group and others have demonstrated an improved regeneration for sensory neurons as well (Brushart et al., 2005; Geremia et al., 2007; Singh et al., 2012; Wong et al., 2015). Recently, Elzinga and colleagues have examined the effects of the ES paradigm for delayed nerve repair showing an effective repair as in the case of immediate treatment (Elzinga et al., 2015). On the other hand, other studies conducted in the CNS have shown that ES promotes sprouting but not regeneration. Previous work have shown that following pyramidotomy to the corticospinal tract (CST), a daily application of ES to the motor cortex (M1), for a period of 10 d, caused robust sprouting of CST axons in the impaired side (Brus-Ramer et al., 2007; Carmel et al., 2010; Zareen et al., 2017). The duration of daily stimulation was depended on the type of stimulation and could range from 6 h of multipulse stimulation (MPS) to 30 min of intermittent theta burst stimulation (iTBS). The observed difference between sprouting and regeneration following ES cannot be automatically attributed to the difference between the CNS and the PNS, nor to the difference between the types of the stimulation.

Although experimental results of the regeneration of populations of axons are promising, the data regarding the precise effect of ES are still controversial and to date the effect of ES on single neuron behavior has not been shown. Moreover, due to neuronal heterogeneity, different cell types with different functions and targets may respond differently to the same ES protocol (Hathway et al., 2009). Previous studies have showen that, intrinsically, different types of neurons demonstrate different regeneration capabilities (Duan et al., 2015; Hu et al., 2016; Jacobs et al., 1997; Wu et al., 2007). Hence, observations of selected identified neurons may significantly reduce biological noise resulting from population averaging, and may allow for detailed characterization of the axon response to the ES.

Simple model systems such as that of invertebrates that allow the analysis of identified cells within the intact arrangement may be beneficial. Despite the differences in complexity between the vertebrate and the invertebrate nervous systems, the latter has been proven to be useful for understanding basic mechanisms related to neurophysiology, development, and regenerative biology (Baylor and Nicholls, 1971; Brenner, 1974; Ready and Nicholls, 1979; Bellen et al., 2010; Kandel, 2012). The CNS of the adult leech has been a useful model for studying these topics over 50 years (Kuffler and Nicholls, 1966; Macagno, 1980; Kristan et al., 2005; Firme et al., 2012; Tomina and Wagenaar, 2017; Puhl et al., 2018). It is finite and relatively simply interconnected. It is comprised of a head ganglion, 21 mid-body ganglia, and a tail ganglion that are joined by two lateral connectives and one smaller medial connective called Faivre’s nerve (Coggeshall and Fawcett, 1964). Each mid-ganglion, except #5 and #6, contains ∼400 highly accessible neurons (Macagno, 1980). The neurons of the adult leech CNS can be unambiguously identified based on a typical size, spatial location and electrical properties (Cohen et al., 2019). Importantly, the leech CNS undergoes spontaneous and relatively fast repair following injury (Macagno et al., 1985; Chiquet and Nicholls, 1987; von Bernhardi and Muller, 1995; Wang et al., 2005; Mladinic et al., 2009). Furthermore, the involvement of microglial cells in the regeneration process following neuronal injury was first shown in this model (Sieger and Peri, 2013), and it was used to elucidate the signaling pathways that mediate their migration and crosstalk with the damaged neurons (Morgese et al., 1983; McGlade-McCulloh et al., 1989; Chen et al., 2000; Duan et al., 2003, 2009; Ngu et al., 2007; Schikorski et al., 2008; Salzet and Macagno, 2009; Croq et al., 2010; Samuels et al., 2010; Tahtouh et al., 2012; Arafah et al., 2013; Le Marrec-Croq et al., 2013; Drago et al., 2014).

In this study, we examined the promoting impact of brief ES on the regeneration of the leech CNS. We specifically focused on one type of identified neurons, the Retzius cells. These are neuromodulatory neurons, the largest within each ganglion, sending their axonal projections to adjacent ganglia through the connectives. They are involved in various leech behaviors including swimming, crawling and local bending (Tomina and Wagenaar, 2017). As a comparison, we examined the P cells that are mechanosensory neurons, responding to moderate pressure to the leech ventral (Pv) or dorsal (Pd) skin by specific spiking patterns that encode different spatial and temporal features of the stimuli (Nicholls and Baylor, 1968; Pirschel and Kretzberg, 2016). We examined the morphology and activity of both types (Retzius and Pd cells), within the intact adult leech CNS, following an injury inflicted by a partial cut of the ganglia chain *ex vivo*. The identified neurons were labeled and their regeneration was assessed over 72 h, with and without ES. In addition, we analyzed the ES-induced response of microglial cells within the injured connectives.

## Materials and Methods

### Animals and dissection

**Figure 1.**
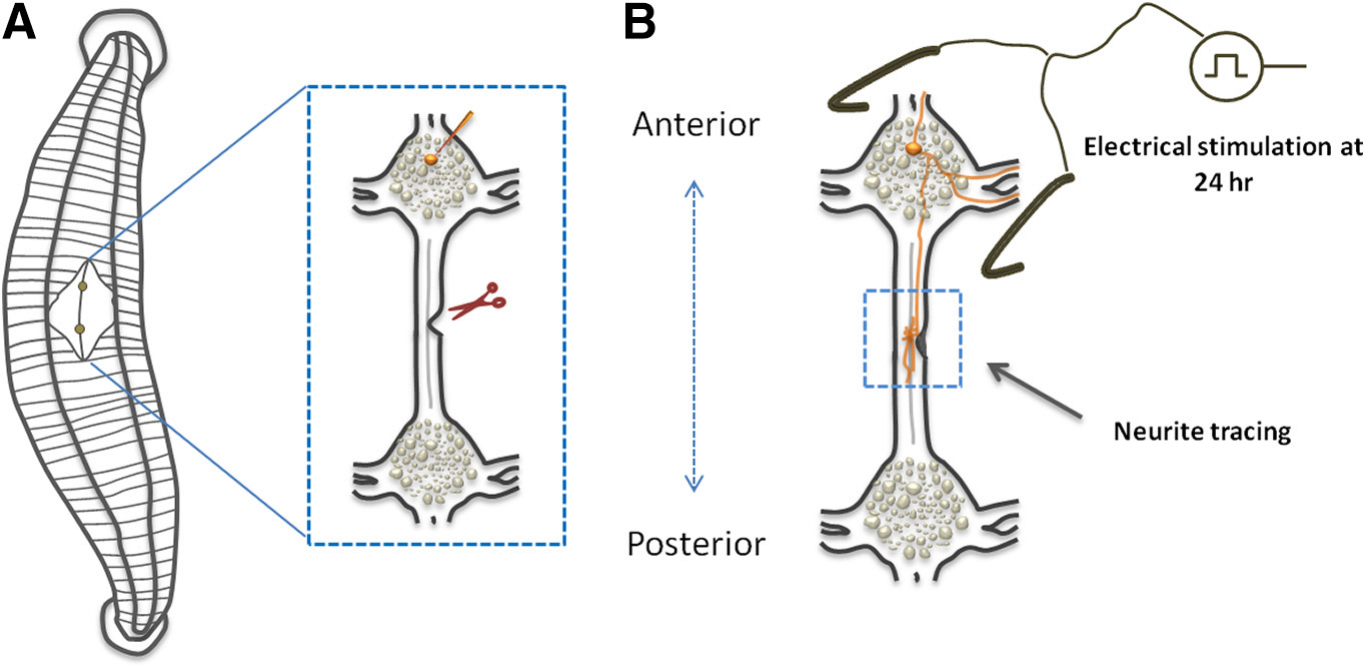
Schematic overview of the experimental procedure. ***A***, Leech dissection was performed for the isolation of ganglia chain (enlarged insert). An identified neuron was fluorescently labeled by microinjection (orange) and a partial cut was made to one of the two connectives. ***B***, Ganglia chains were left to recover either spontaneously, or following a brief ES applied 24 h after injury. Newly formed axonal branches were traced at the injury site 72 h after injury (blue dashed square).

Ganglia chains were isolated from the CNS of adult medicinal leeches *Hirudo medicinalis* (Fig. 1*A*). All leeches are hermaphrodites. Leeches were anesthetized on ice for 30 min before dissection and were pinned, dorsal side up, to a layer of Sylgard on the bottom of the dissection chamber. Dissection was made according to an established procedure (Titlow et al., 2013). Briefly, a longitudinal cut was made through skin and muscle layers along the dorsal midline of the leech to expose the nerve cord and segmental ganglia. Short segments of CNS chains comprised of two adjacent ganglia joined by connective tissue, were then dissected and moved to a Sylgard-148 Petri dish containing 4 ml of room temperature enriched Leibovitz medium (L15 medium supplemented with 6 mg/ml glucose, 0.1 mg/ml gentamycin, 2 mm/ml glutamine, and 2% fetal bovine serum). Next, ganglia chains were pinned on the Sylgard layer, ventral aspect up. After pinning the chains, we specifically labeled the anterior right side Retzius cells in accordance with a description below. For the P cell experiments, we labeled the Pd cell on the injured side. The chain orientation was kept the same for all chains. Partial axotomy was then performed by cutting one of the two connective tissues (i.e., the connective that includes the axonal projection of the labeled cell) with fine scissors. Finally, the L15-enriched medium was replaced with fresh medium, and the injured ganglia chains were left to recover in the 25°C incubator up to the ES part.

### Neuronal labeling by intracellular injection

Sharp injection micropipettes were pulled from borosilicate glass capillaries of 1-mm external diameter and 0.75-mm internal diameter using a p-1000 micropipette puller (Sutter instruments). A resistance of 15–25 MΩ was obtained by filling the micropipettes with 3 m potassium acetate. To visualize a single regenerating axon within the CNS, we examined different fluorescent dyes based on previous reports of the leech nervous system and other invertebrate neurons that have shown effective tracing of fine neuronal structure (Fan et al., 2005; Shefi et al., 2006). Dextran amine conjugated dyes (Alexa Fluor and Fluro-Ruby) were found to be ideal for our purpose. Hence, for labeling, micropipettes were filled with either 3–5% Alexa Fluor 488/568 (catalog #D-22910/D-22912) or 3–5% Fluoro-Ruby (catalog #AG335). Since the Retzius cells are the largest pair of cells situated on the mid-ventral surface of each segmental ganglion and are characterized by a typical spontaneous electrical activity, they were highly accessible and very easily identifiable. P cells, on the other hand, may be confused with other cells in their environment, and their electrical activity must be monitored for unambiguous identification. By using a micromanipulator, we positioned the filled micropipette in the desired cell, impaled it, and injected the dye iontophoretically with a positive current of 15–35 nA for 30 s to 1 min (Movie 1). This short-time injection was found to be sufficient to label the desired cell for further analysis. Using the same micropipette, we monitored the neuronal activity of the cell before and after dye injection to verify cell viability along the procedure. Signals were amplified (molecular devices multi clamp 700B), filtered and digitized by an analog-to-digital board Digidata 1400A (Axon Instruments).

Movie 1. Retzius labeling by intracellular injection. Injection electrode was filled with 5% Fluoro-Ruby and positioned toward the Retzius neuron. The neuron identified according to its typical location and size. Following cell penetration, we applied positive current of 15 nA to move the dye out of the electrode into the cell. 10.1523/ENEURO.0030-19.2020.video.1

### ES

Twenty-four hours after injury (day 1), a single ES was applied to the experimental chains (Fig. 1*B*). The control chains received sham ES. In order to precisely control the electrode progression toward the stimulation location, we designed and constructed a generic light aluminum electrode holder that allowed the coupling of the platinum iridium parallel bipolar hook electrodes (FHC - PBAA08100) with a three-axis motorized micromanipulator. We used the micromanipulator to place the electrodes next to the ganglia chain near the ganglion. Specifically, cathode and anode were placed at opposed sides of the ganglion. The electrodes were connected to a function generator and an ES of continuous 20-Hz square wave, positive polarity, and amplitude of 500 mV/mm, 1 V/mm, or 3 V/mm was generated for 30 min. Subsequently, the electrodes were removed and the medium was replaced with a fresh sterile medium. The ganglia chains were allowed to recover in a 25°C incubator for additional 48 h.

### Morphometric analysis

Newly formed axons were detected at the injury site 72 h after connective partial cut. For each ganglia chain, Z-stack images from the injury site were acquired with a fluorescence microscope (Leica Z16-APO) equipped with the LAS Montage Module for acquiring a series of Z-Stack images and with the appropriate filters of FITC and TRIRC. We measured the morphometric parameters of the newly formed branches with or without ES. These included total branching tree length and the percentage of nerve processes succeeding in crossing the injury site for each regenerating neuron. To measure neural length in the 3D tissue, we used the simple neurite tracer plugin of the Fiji ImageJ software (National Institutes of Health; Meijering et al., 2004), which enables semiautomatic neurite tracing and length measurements for the fluorescently labeled regenerating axons. To measure the percentage of nerve processes which crossed the injury site, we set the point at which the axon had been cut as a reference point and calculated the proportion of the total branches length that crossed this point out of the total branching tree length of the entire regenerated axon.

### Microglia distribution analysis

Ganglia chains were isolated, pinned on the Sylgard layer, and were subjected to a partial cut injury as described earlier (see also Fig. 1*A*). Injured ganglia chains, experimental and control, were placed in the 25°C incubator to allow microglial cell migration toward the injury site, as described previously. Twenty-four hours after injury, an ES at 20 Hz for 30 min was provided only for the experimental ES group, whereas the control group underwent sham stimulation. L15 medium was then replaced, and chains were returned to the incubator for additional 24 h. Next, 48 h after injury, cell nuclei were stained with SybrGreen fluorescent dye, to observe cell distribution along the chains. To note, some of the nuclei, a minority of the entire population, were those of the perineurial sheath cells and another one nucleus belonging to the single glial cell related to the connective. Since perineurial sheath cells do not move following injury, a change in the number of cells is mainly caused by a change in the microglial cell population (as noted by McGlade-McCulloh et al., 1989). A series of fluorescent Z-stack images were acquired from two regions of the connective: by the ES site and by the injury site. Cells were counted manually by using Fiji ImageJ software, and the total number of cells for each condition was obtained and compared.

### Experimental design and statistical analyses

Measurements of the investigated parameters are summarized in bar plots. Values were expressed as a mean ± SE. The unpaired two-tailed and one-tailed (when direction of outcome is predictable) Student’s *t* test were used to determine statistical significance; *p* < 0.05 was considered statistically significant. The number of samples and the statistical analysis for each panel, including specific *p* values, are indicated when appropriate in each figure legend in the results section.

## Results

### Regeneration of single cells within *ex vivo* ganglia chain following a cut model

Illustration of the model system and the experimental procedure can be seen in Figure 1. The two steps of the preparation include the ganglia chain isolation and injury (Fig. 1*A*), and the regeneration following brief ES (Fig. 1*B*).

**Figure 2.**
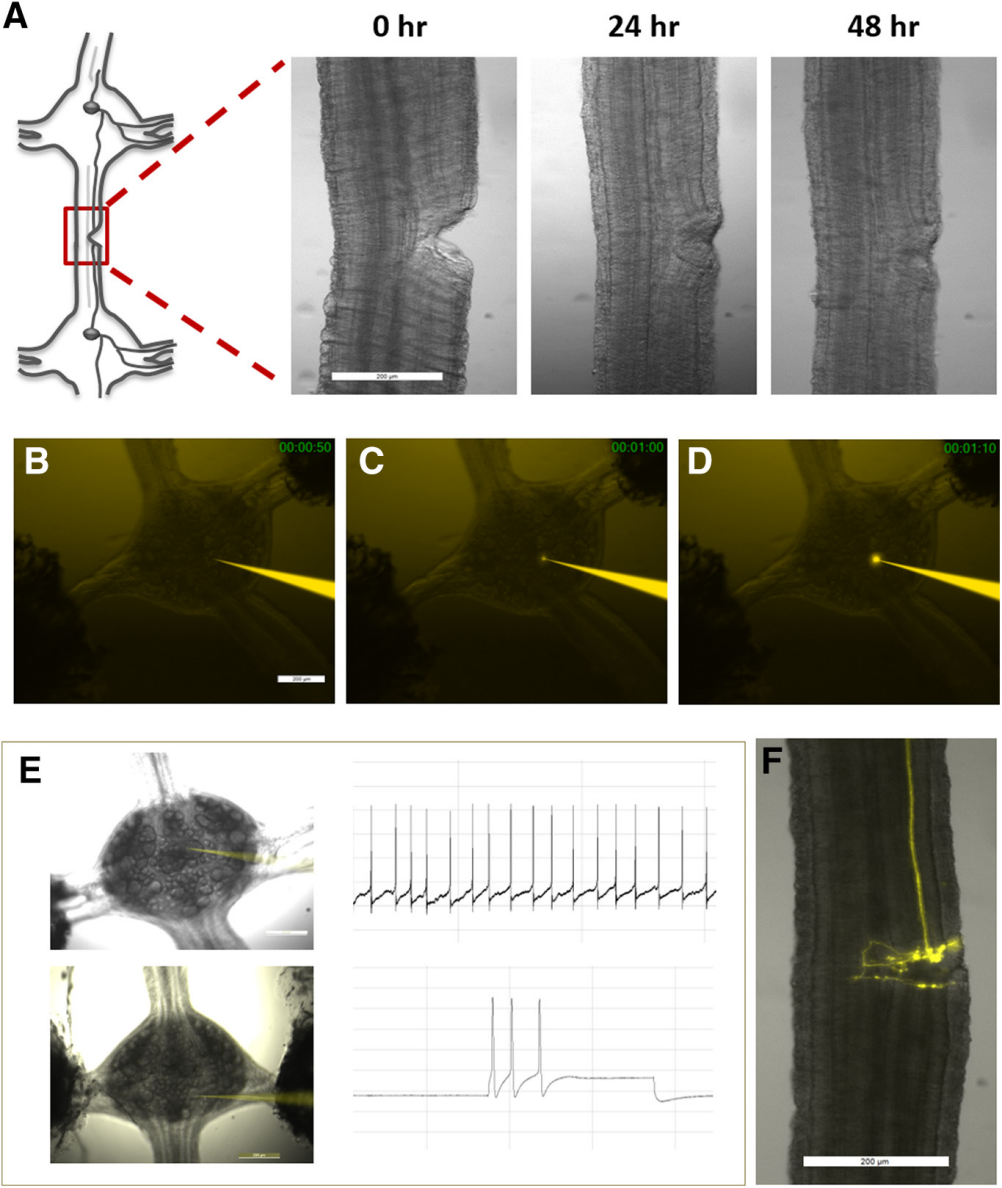
Tracing newly regenerated axonal branches of an identified neuron within the recovering leech CNS. ***A***, Spontaneous regeneration process of the connective tissue following partial axotomy of one of the connectives. ***B–D***, Microinjection of a single neuron with Fluoro-Ruby for a period of up to 1 min. Time lapse images demonstrate the procedure. ***E***, Retzius cell (upper panel) and P cell (lower panel) identified according to their typical location, size, and characteristic electrical activity pattern. The dye-filled recording electrode can be seen in bright orange in both images. ***F***, Fluorescent dye demonstrates the elaborated trajectory of the newly regenerated axonal branches of the Retzius neuron following an injury. Scale bar = 200 μm (***A–F***).

The spontaneous regeneration of the injured ganglia chain can be seen in Figure 2*A*. A recovery of the connective tissue could be detected already 24 h after injury. Figure 2*B* shows a recording electrode (bright orange) impaling the soma of a large Retzius cell before labeling. After impaling the cell, dye was injected iontophoretically (Fig. 2*B–D*). To verify neuronal viability following the injury and the labeling procedure, we monitored the spontaneous spike activity of the injected Retzius neurons or evoked potentials in the injected P cells before and after dye injection (Fig. 2*E*). All neurons retained their typical spike activity following the procedure. Figure 2*F* demonstrates the newly regenerated axonal branches of the Retzius neuron 72 h after injury, revealing an elaborated growth at the injury site with a complex branching tree pattern.

### Comparison between anterior and posterior cell spontaneous regeneration shows no CNS polarity

**Figure 3.**
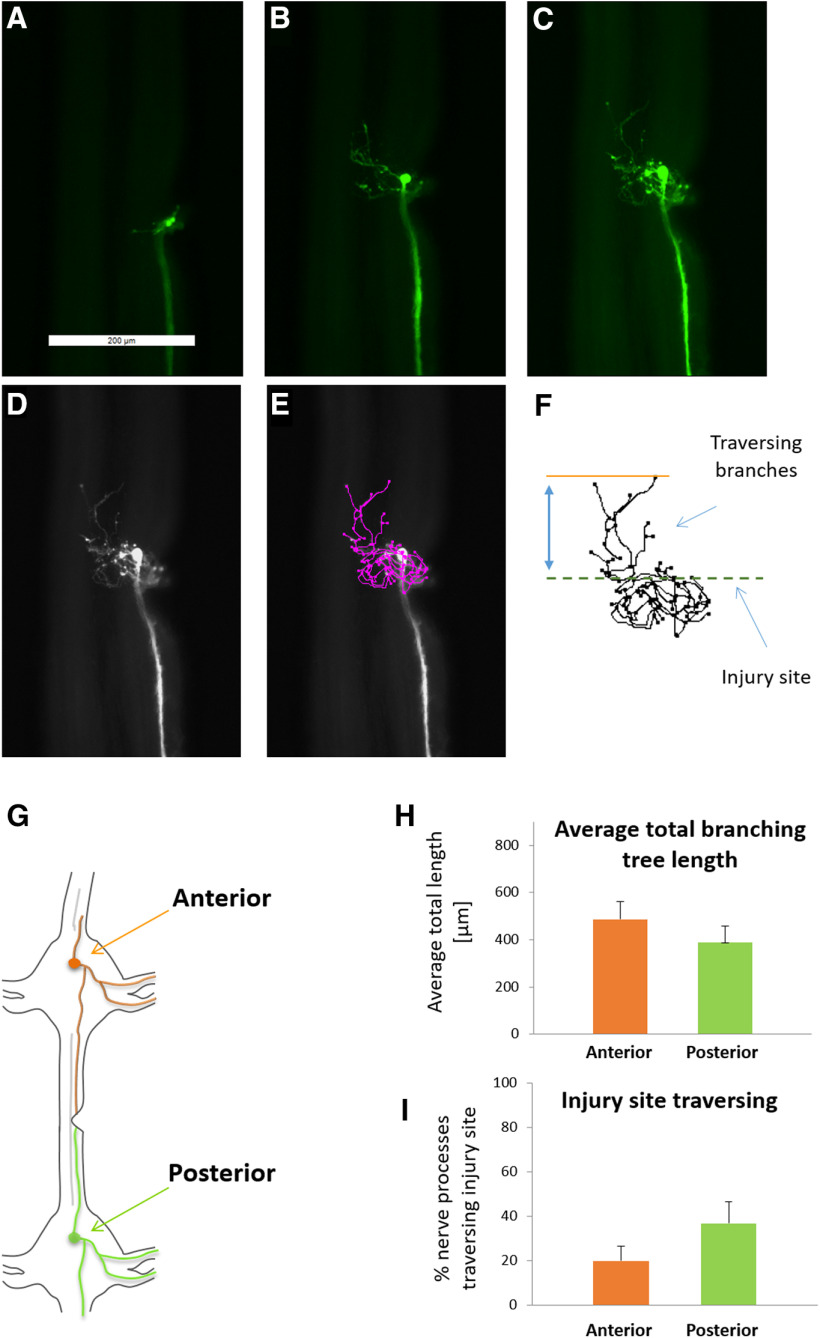
Antero-posterior polarity during CNS spontaneous regeneration. ***A–C***, Fluorescence images of a single labeled Retzius neuron’s newly formed branches at time points 24, 48, and 72 h after injury, respectively. ***D***, ***E***, Morphologic measurements method. Neurite tracing of the newly formed branches (pink) was performed by the semi-automated simple neurite tracer plugin. ***F***, FIJI drawing representing an example of typical newly formed axonal branches. The drawing shows the relative portion of mass that crosses the injury site out of the total newly branching tree. ***G***, Illustration of antero-posterior polarity examination. Retzius cells were microinjected either at the anterior (*n* = 16) or the posterior (*n* = 19) ganglion. Cells that exhibited successful regeneration after 72 h (i.e., cells with >150 μm newly regenerated axonal branches) were further analyzed. ***H***, Averaged total lengths of the newly formed axonal branches (no significant difference). ***I***, Percentage of axonal branches crossing successfully the injury site (no significant difference). *N* = 15 per group, two-tailed unpaired *t* test. Scale bar = 200 μm.

To characterize the spontaneous regeneration process of neurons that have not received ES, we stained the neurons, traced the regenerating trajectories and followed the recovery up to 72 h after the cut. Figure 3 shows a typical regeneration process in a single neuron as imaged 24, 48, and 72 h after injury. A dynamic axonal regeneration can be seen, indicating that the neuron remained viable. To evaluate functional regeneration, we calculated the proportion length of the branches that crossed the injury site (traversing branches) out of the total branching tree of the regenerated axon (Fig. 3*F*). Next, we examined whether there is a distinguishable antero-posterior polarity within the leech CNS that may affect the spontaneous injury site traversing. We obtained axonal tracing of regenerating Retzius neurons from both anterior and posterior ganglia (relative to cut location; Fig. 3*G*). No significant difference between the two groups was found in the average branching tree length (487 ± 75 vs 389 ± 70 μm, respectively; Fig. 3*H*) nor in the percentage of axonal branches mass succeeding in crossing the injury site (20 ± 7 vs 37 ± 10, respectively; Fig. 3*I*). The results indicate that there is no CNS polarity that may affect the spontaneous injury site crossing with respect to Retzius spontaneous regeneration.

### Typical spontaneous regeneration patterns for identified sets of neurons

**Figure 4.**
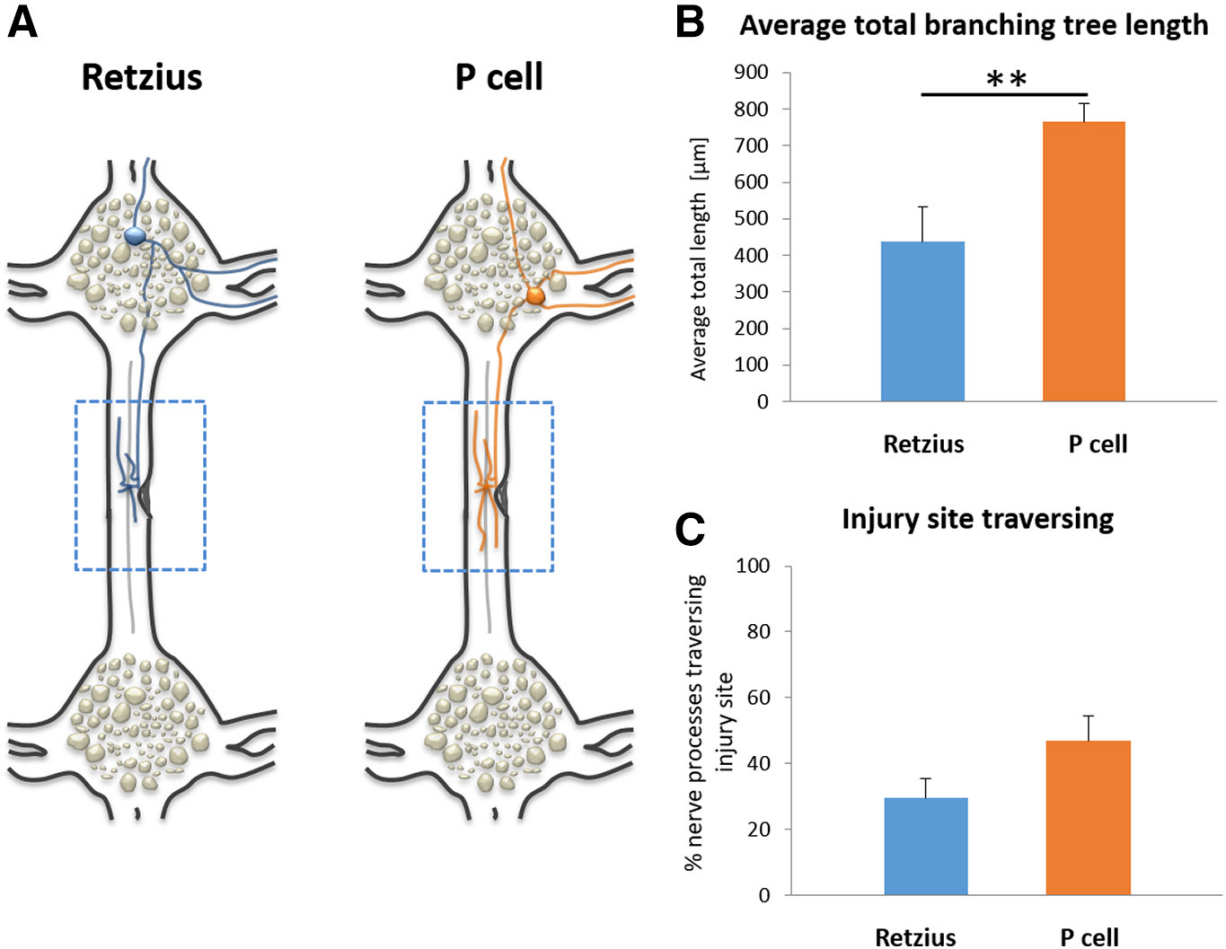
Different types of neurons demonstrate different spontaneous regeneration. ***A***, Illustration of the single Retzius and P cell tracing. ***B***, Total branching length of spontaneously regenerated Retzius and P cells. Two-tailed unpaired *t* test; *p = 0.0019. ***C***, Injury site traversing of spontaneously regenerated Retzius and P cells.

To evaluate whether different types of neurons may exhibit different regeneration features following injury, we compared Retzius to P cells spontaneous regeneration. P cells from both anterior and posterior ganglia (*n* = 18) were compared with the Retzius neurons, also taken from anterior and posterior ganglia (*n* = 29). The different types of cells demonstrated different basal behavior. The results indicate that P cells were able to significantly regenerate larger branching tree compared with the regenerating Retzius neurons (764 ± 96 vs 436 ± 51 μm, respectively; Fig. 4*B*). In addition, P cells demonstrated a relatively higher rate of injury site crossing compared with the Retzius neurons (47 ± 8 vs 29 ± 6, respectively; Fig. 4*C*). However, this effect was not statistically significant. These data clearly show that different types of cells may present different responses and that by exploring single neurons this information becomes visible.

### Injury site traversing by regenerative axons significantly increased following brief ES for Retzius neurons

**Figure 5.**
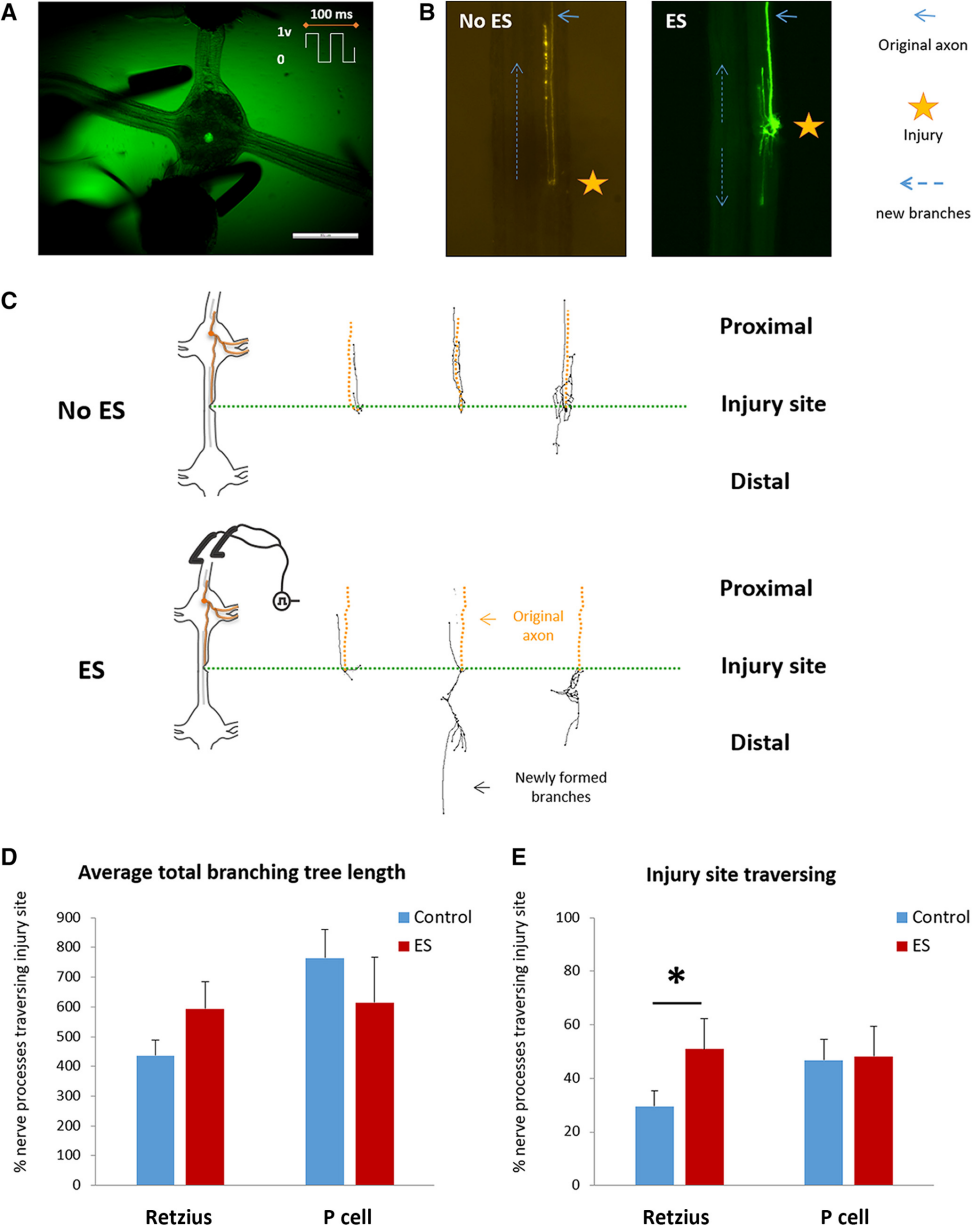
Morphologic analyses for Retzius and P cells regeneration with and without ES. ***A***, Fluorescence image of the ganglion stimulation. The bipolar hook electrode was positioned in the desired location so that the ganglion was situated in the center. The pattern of the ES is displayed at upper right corner. ***B***, Fluorescence images of the Retzius cell’s newly formed branches at 72 h, with and without ES (green and orange, respectively). ***C***, Representative examples of three ganglia chains from a single leech for each condition. Upper panel, Chains with no ES treatment. Lower panel, Chains treated with ES. The axonal tracing drawings of the newly formed branches were placed on an artificial line that illustrates the relative position of the injury site (green dotted line). The original axon is marked by the orange dotted line and the newly formed branches are in black. The portion of the axonal tree located under the green dotted line is the mass that crossed the injury site. ***D***, Total branching length of Retzius and P neurons in non-stimulated (left bar) and stimulated (right bar) chains. ***E***, Percentage of injury site crossing of Retzius and P neurons in non-stimulated (left bar) and stimulated (right bar) chains. One-tailed unpaired *t* test; *p = 0.041; *n* = 40 (Retzius cells) and 26 (P cells).

To examine the effects of ES on the regeneration we applied a brief ES (20 Hz, 1 V/mm) to the ganglia chain 24 h after injury (Fig. 5*A*) and quantitatively analyzed the morphology of the labeled neurons after another 48 h. This intensity was chosen according to an earlier study that demonstrated positive effect of ES on neurite outgrowth of goldfish retinal explants (Ou et al., 2012). Based on previous studies showing improved regeneration following ES in comparison to spontaneous regeneration (Borgens et al., 1981; Nix and Hopf, 1983; Borgens, 1999; Al-Majed et al., 2000a; Brushart et al., 2005; Gordon et al., 2009; Carmel et al., 2010; Ou et al., 2012; Singh et al., 2012; Elzinga et al., 2015; Wong et al., 2015; Gordon, 2016; Zareen et al., 2017; MacEwan et al., 2019), we compared the ES-treated versus non-treated cells (using one-tailed *t* test) in analyzing the ES effect. Examples of typical regeneration patterns of Retzius cells with and without ES are shown in Figure 5*B*. Axonal tracing drawings of newly formed branches demonstrate that larger mass of branches crossed the injury site and were directed toward their original target, the distal ganglion, following an ES as compared with spontaneous regeneration of the unstimulated group (Fig. 5*C*).

Comparison between the average branching tree length of regenerating Retzius neurons with and without application of ES showed that there is no significant difference between the groups (593 ± 90 vs 436 ± 51 μm, respectively; Fig. 5*D*). However, a comparison between branches’ directionality of regenerating Retzius neurons with and without application of ES showed a significant increase in percentage of axonal branches (length) which successfully crossed the injury site. For stimulated Retzius cells, the regenerating branches mass that crossed the injury site increased significantly to an average percentage of 51 ± 11%, whereas the unstimulated Retzius cells showed an average percentage of only 29 ± 6% (Fig. 5*E*). This result reflects an almost two-fold increase, similar to the increase reported for populations of rat sensory and motoneurons (Elzinga et al., 2015). To examine the effect of the ES amplitude on the ability of axons to cross the injury site, we repeated the procedure with higher (3 V/mm) and lower (500 mV/mm) amplitudes. Application of ES with lower amplitude of 500 mV/mm (*n* = 6) led to similar statistics as for the amplitude of 1 V/mm (61 ± 10% vs 51 ± 11%, respectively). However, the application of higher ES amplitude of 3 V/mm resulted in an immediate observable damage to the ganglion, reflected in a distortion of its shape. In an attempt to clarify the nature of the axons outgrowth with and without ES, we analyzed the number of branches for each condition. The average number of branches was similar for cells with and without the electrically stimulation (7.6 ± 1.0 vs 6.3 ± 0.7 branches, respectively). This result suggests that the ES affect the outgrowth directionality without changing the level of axon sprouting.

The analysis of P cells morphology led to different growth behavior than the Retzius cells. Application of ES had no effect of the rate of injury site crossing that was close to 50% even without the ES stimulation (48 ± 11 vs 47 ± 8, for stimulated and non-stimulated, respectively; Fig. 5*E*). In addition, there was no significant difference in P cells branching tree length with or without the brief ES (614 ± 154 vs 764 ± 96, respectively; Fig. 5*D*).

### Injured ganglia chains present different microglial cell distribution following brief ES

**Figure 6.**
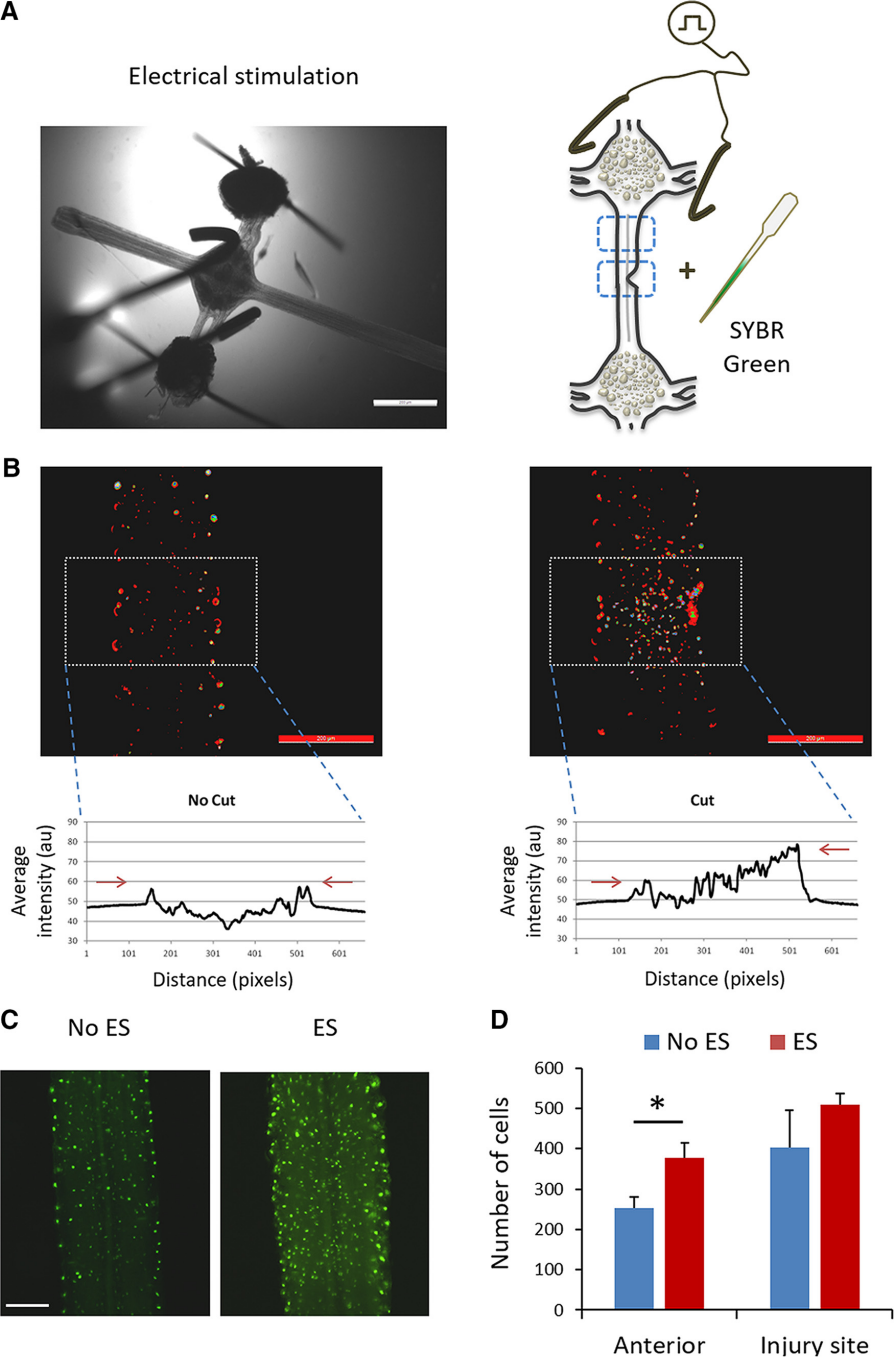
Electrical effect on leech microglia. ***A***, Illustration of the experimental procedure demonstrating cell nuclei staining with SYBR Green in the vicinity of the location of ES and at the region of interest for analysis (blue dotted rectangles). ***B***, Measurement of SYBR Green intensity profiles in cells within the leech connective. The fluorescence intensity (*y*-axes) measured from left to right along the rectangular regions (*x*-axes) were plotted. Cell distribution intensity profile shows accumulation of cells at the injury site and increased values for the side that matches the injury area as compared with relatively uniform values in the uninjured chain. The images show the cells after pseudo-color processing. Blue and green represents high intensity, red represents low intensity. ***C***, Visualization of SYBR Green-labeled microglia distribution and accumulation following partial cut with and without ES. Fluorescence images show increased number of cells at the anterior region only for the ES group. ***D***, Average number of cells at the two regions of interest with and without ES. Asterisk indicates that the cell number near the ES site (anterior) was significantly increased under brief ES; *p = 0.048, *n* = 3–4 CNS ganglia chains per group, two-tailed unpaired *t* test. Scale bar = 200 μm (***A–C***).

To examine whether brief ES alters the microglial cell response during leech CNS recovery, we labeled the cell nuclei along the connective 48 h after injury and compared the number of cells with that found in the untreated chains. As it has been shown that the nuclei in the connective are mostly those of microglial cells, and that there are no other migrating cells along the connectives other than microglia (McGlade-McCulloh et al., 1989; Samuels et al., 2010), a change in the number of nuclei along the connective can be attributed mainly to a change in the microglial cells population. For each condition we analyzed two regions in the connective: near the ES site, i.e., the anterior region, and close to the injury site (Fig. 6*A*). Figure 6*B* demonstrates a preferential accumulation of glial cells at the injury site, as differential fluorescence intensity with higher values can be seen in the injured chains in comparison to a lower and relatively homogeneous fluorescence intensity observed in the non-injured chains. As for the anterior region of the chain, close to the ES site, a higher number of cells was detected in the electrically stimulated injured chains compared with the non-treated ones. As shown in Figure 6*C*,*D*, there was a significant difference in the total number of cells counted at the anterior region between the two groups (380 ± 40 vs 250 ± 20, respectively).

## Discussion

Simple model systems of invertebrates are beneficial for studying the basic principles of nervous system regeneration (Baylor and Nicholls, 1971; Ready and Nicholls, 1979; Yanik et al., 2004; Yan et al., 2009). One such preparation is the CNS of the medicinal leech, *H. medicinalis*, in which spontaneous regeneration that leads to functional recovery can occur. Using a cut injury model of the nervous system of the medicinal leech, we were able to analyze the regeneration process of identified neurons at the single cell level with and without ES. For each ganglia chain, we anterogradely labeled single cell body. We analyzed specific cells, the Retzius neurons, which are paired serotonergic neurons with the largest somas in the ganglion, and traced the branching morphology at the injury site for up to 3 d. Our results show that Retzius neurons exhibit successful spontaneous regeneration within this period; however, the vast majority of the regenerating branches fail to regrow across the lesion site and most of them turn backwards. To examine the advantage of labeling specific type of neurons, we tested the spontaneous regeneration behavior of a second type of neurons, the P cells. We found a distinct growth pattern for each cell type with higher level of efficient injury site crossing for the P cells. Previous studies already demonstrated different regeneration capabilities to different neuronal subtypes. Duan et al. (2015), for example, showed that following an injury to the mouse optic nerve, and subsequent treatment that elicits regeneration, only one specific subtype of the retinal ganglion cells (RGCs) out of 11 subtypes, the αRGCs, was accounted for nearly all the observed regeneration. In the *Caenorhabditis elegans* several mechanosensory neurons such as the PLM, ALM, and AVM show robust regeneration (Wu et al., 2007), while other sensory neurons, such as the AFD and AWC, show no regeneration following axotomy (Chung et al., 2006; Gabel et al., 2008). In the sea lamprey, following an hemisection of its spinal cord, some neurons, such as the Mauthner, I1, B1, and B3 neurons, display poor regenerative capacity, while others, such as the M4, I3-I6, B2, and B6, show high regenerative capacity (Jacobs et al., 1997). The differences in the regeneration capabilities are attributed to intrinsic differences between neuron types (Jacobs et al., 1997; Shifman and Selzer, 2007; Chen et al., 2016; Chen and Shifman, 2019), which may also mediate different regenerative responses to the same ES.

Previously, using different models has shown that axonal regeneration and preferential re-innervation following brief ES is accelerated (Al-Majed et al., 2000a; Gordon et al., 2009; Singh et al., 2012; Elzinga et al., 2015).

Analyzing neurons that can be unambiguously identified allowed us to quantify the effect of the ES per cell type. Following ES, in contrast to the backward regrowth typically exhibited along the Retzius spontaneous regeneration, the regenerated axonal branches of the Retzius cells changed their directionality without changing the total branching tree length. We found that brief ES affected the typical tree directionality and promoted its ability to cross the injury site. Branches that succeeded in crossing the injury site were able to keep growing toward the adjacent target ganglion. This approach revealed a way to overcome the limited regeneration following a cut-induced injury, which acts as a more rigid barrier for axons to cross as compared with other injuries such as crushes (Macagno et al., 1985). Indeed, Macagno et al. (1985) have demonstrated an extremely poor ability of T cells (mechanosensory neurons) to cross the injury site following a cut compared with crushing the connective. In addition, they found that the T cells in a cut model typically reverse the regrowth direction toward the original ganglion, as we have noticed in our experiments for the Rz neurons. However, this phenomenon is not the same for all neurons. We examined another set of mechanosensory neurons (P cells) and found that half of the axonal tree could cross spontaneously the injury site. These results show a typical regrowth pattern and are in accordance with pervious results of another model system, the sea lamprey, that showed the tendency of some axons to regenerate across the lesion, rather than loop backward (Mackler et al., 1986; Lurie and Selzer, 1991). The limited improvement of injury site crossing of P cells with ES application may represent a ceiling effect, raising an interesting question for future study. Thus, the effect of ES, as we showed here, may contribute to an efficient and functional regeneration pattern for cells that otherwise tend to turn backward at the injury site, improving the natural effectiveness of regeneration.

To rule out an immanent anterio-posterior polarity within the adult ganglia chain, which may lead to the backward regeneration trend, we examined the spontaneous regeneration pattern for both anterior and posterior Retzius neurons (relative to the injury site). No significant difference was detected regardless of whether the neuron was regenerating from the anterior ganglion to the posterior or vice versa. This shows that the tendency to loop back is not affected by the position of injured neuron and this behavior is a result of the regeneration after injury. This result strengthens our conclusion that the spontaneous recovery, after a partial cut injury, has a backward-regeneration trend and that the brief ES interferes with this pattern to some extent, leading to a more efficient pattern of regrowth.

Previous studies suggested that action potentials generated by the ES are transmitted back to the soma of the neurons, potentially promotes upregulation of BDNF and other neurotrophic factors and their receptors and are essential for enhancing axonal growth following stimulation (Al-Majed et al., 2000a,b, 2004; English et al., 2007; Geremia et al., 2007; Elzinga et al., 2015; Gordon, 2016). The blockade of the retrograde transmission of these action potentials by TTX prevented the positive effect of the ES on the axonal outgrowth. So far, none of the canonical neurotrophic factors (NGF, BDNF, CD3/4, and GDNF ligands) have been found in the invertebrate nervous system (Palgi et al., 2009). Therefore, we seek for other evolutionary conserved mechanisms that can be involved in mediating the ES effect. Different models, including mammalian nervous systems, revealed that glial cells are activated following nerve injury (Schwartz, 2003; Davalos et al., 2005; David and Kroner, 2011). In addition, in diverse models including mice and rats, it has been shown that microglial cells respond rapidly to any minor neuropathological change (Kreutzberg, 1996; Hanisch and Kettenmann, 2007). In the leech, microglial cells recruitment at the injury site is known to be involved in the regeneration process. Previous studies have suggested that, within the leech CNS, cells start moving toward the injury site within minutes after injury, reaching peak level within 24 h and then decline slowly for weeks after injury (Morgese et al., 1983; McGlade-McCulloh et al., 1989; Le Marrec-Croq et al., 2013). We, therefore, also analyzed the migration and distribution of microglial cells in injured ganglia chains, electrically stimulated and non-stimulated. Following the injury, we observed greater accumulation of microglial cell close to the stimulating area (between the anterior ganglion and the injury site) in ganglia chains that received ES. At the injury site, a considerable accumulation of cells was observed for both cases, but no significant ES-induced difference was detected between those groups. Our findings that brief ES increased the axonal crossing of the injury site and affected microglial cells’ migration lead to the conclusion that the modifications in axonal growth direction following a brief ES might be mediated by ES through differential microglial cells distribution. Activating microglial subpopulations by different factors has been reported previously in the leech (Croq et al., 2010; Tahtouh et al., 2012) and also in mammals (Hanisch and Kettenmann, 2007), leading to neuroprotective or neurotoxic effects. Our findings of amended growth pattern and, most importantly, efficient neuronal regeneration triggered electrically are intriguing with high potential for promoting neuronal repair. Moreover, the observed brief ES-induced supported functional regrowth, which was able to overcome the physical obstacle formed by a scar, opens new possibilities for future therapeutics.

## Synthesis

Reviewing Editor: Jeffery Twiss, University of South Carolina Decisions are customarily a result of the Reviewing Editor and the peer reviewers coming together and discussing their recommendations until a consensus is reached. When revisions are invited, a fact-based synthesis statement explaining their decision and outlining what is needed to prepare a revision will be listed below. The following reviewer(s) agreed to reveal their identity: Michael Selzer. Note: If this manuscript was transferred from JNeurosci and a decision was made to accept the manuscript without peer review, a brief statement to this effect will instead be what is listed below.

en-TNWR-0030-19X

Synthesis statement

The authors examined the effect of electrical stimulation (ES) on single neuron regeneration and on microglia migration in the leech nervous system. They present evidence that 20 Hz electrical stimulation for 30 min increased the number of branches sprouting from cut axons of leech Retzius cells, and increased the proportion of those branches that traversed the injury site (as opposed to the usual unstimulated pattern of turning backwards), but did not increase the average length of those branches. The basic findings are interesting and well illustrated, but there are a number of concerns. The effect of electrical stimulation on axon regeneration has been investigated in other systems, albeit not at the single neuron level. The authors show a difference in microglia migration in response to injury and electrical stimulation which may be of significant importance, especially because the leech system is well suited for the study of microglia and their effect on axon regeneration (Ngu, Sahley et al. 2007, Le Marrec-Croq, Drago et al. 2013). While we appreciate the significant effort required to produce single neuron data in this system and the power of the experiments in general, the lack of mechanism, and concerns about the data presented prevent us from recommending this paper for publication at this time. Also there are concerns on the numbers of animals tested and effect sizes that need to be addressed.

Major concerns:

1. It is not clear whether the electrical stimulation is meant to activate action potentials in the Retzius cells or has some other effect, such as that on microglial migration or proliferation. The authors state that one of the main reasons to explore the effect of ES on single neurons is the fact that different neurons may respond to ES in different ways when injured. Thus, repeating these experiments on at least one other type of neuron in the same system would significantly strengthen the manuscript and address their point on distinct responses in different neurons. The authors should at least acknowledge and discuss the limitations of studying a single neuron population, as well as put this in the context of other studies mentioned below. Further, they need to specify the polarity of the stimulation used, and in their discussion, they should clarify whether the papers in the literature that they cite deliver stimuli to activate neuronal action potentials or have a different action, such as with DC current.

2. The authors only use one paradigm of ES to the entire ganglion and do not explain the rationale for this choice. Stimulating with 20 Hz, 1V over a period of 30 min seems aggressive and the sham control does not specifically address this concern. The authors should show the effect at least one additional ES paradigm. Whether the effect is positive or negative in comparison to the 20 Hz, 1V x 30 min stimulation, it will strengthen the manuscript and may give more insight into mechanisms (which is lacking in this in this submission).

3. The authors have not made the distinctions between sprouting, regeneration, axon retraction and other morphological aspects of response to axotomy that are needed to frame their results in constructive context; analyses of the growth data in this context would strengthen the manuscript. How many axons cross the injury in control vs. ES? Is the difference shown due to very long growth in a few axons or a more sustained growth in multiple axons?

4. The authors point out that similar observations concerning the effects of ES on axon outgrowth have been made by Tessa Gordon, Tom Brushart and colleagues with regard to regeneration of peripheral nerve in mammals. There, the improved specificity of motor and sensory axon innervation of sensory and motor branches of a nerve appears to reflect an increase in the profusion of axon sprouting, which may result in an increased opportunity for selective pruning. The data are less clear and quite controversial in the vertebrate CNS. Probably the most convincing demonstration was that from Jack Martin’s lab, in which electrical stimulation of motor cortex enhanced axon sprouting (but not regeneration) by unlesioned CST axons in the spinal cord after unilateral pyramidotomy. The present authors do not acknowledge that this is a controversial area, and therefore, they miss an opportunity to emphasize why the ability to follow sprouting/regeneration in individual identified axons is so important. The manuscript would be improved if the literature review was a bit more critical and their work were framed in the context of this literature.

5. The finding that electrical stimulation increases both the number of axon branches and the proportion of branches that grow beyond the injury seems inconsistent with their assertion that the total length of axon branch tree did not change, unless the average length of each branch was shorter in the ES-treated axons than in the unstimulated axons. It is possible that we misunderstand the authors’ descriptions, but they should better discuss the nature and implications of their measurements, and whether the distance of regeneration past the injury was greater with ES than without. The axon tracings shown in figures 5 and 6 would seem to suggest that they do, but if that is the case, then ES has an effect not only on the amount of axon sprouting, but also on the distance of regeneration, which is an important distinction.

6. The authors argue that the tendency of axon sprouts to loop backwards does not reflect a polarity in the Retzius cell that favors rostral rather than caudal growth, because axons belonging to Retzius cells whose perikarya are located caudal to the cut also loop back. This is a good point, but it is not expressed clearly in the manuscript.

7. While the increase in regrowth past the injury site in ES is very interesting, the small numbers of animals, moderate effect sizes, and marginal p values increase the overall risk of type one error. This can be addressed by increasing numbers and replicating the findings especially in the experiments in Figures 6 and 7. For example, if one were to compare the posterior growth past injury without ES (37+/-10) to the growth past injury after ES (51+/-11) one might visually see that these data overlap. Additional experiments and analyses will further strengthen the conclusions.

8. The authors observed an increase in cell accumulation in the region of the stimulating electrodes, and suggest that these are microglia that may be promoting the more correct directionality of axon branch growth. The authors assume that all nuclei labeled by SYBR Green belong to microglia because the leech has no astrocytes or oligodendrocytes. To our knowledge, SYBR green is not specific for microglia and the authors did not attempt to label the cells with microglial markers. This should at least be acknowledged the authors, or better yet tested. Moreover, because the electrodes straddle the rostral ganglion and not the lesion, and have their greatest effect on microglial numbers at the site of stimulation, not around the lesion, the effect of ES is to reduce the difference in microglial accumulation between the lesion site and the stimulus site. This would seem to argue against a microglial gradient serving as a guide to push axon sprouts past the lesion. Further, the authors need to consider whether mechanisms previously shown to increase glial recruitment to the injury site are involved in this ES-facilitated regrowth (Ngu, Sahley et al., 2007, J Comp Neurol 503, 101; Le Marrec-Croq, Drago et al., 2013, Clin Dev Immunol 2013, 274019). A test of mechanism would further strengthen the manuscript.

9. Finally, there is a need for English editing in order to clarify the meaning of many passages. One reviewer has provides a copy of the manuscript with suggested revisions in red - this should be accessible in the files section of your eNeuro submission pages as “Reviewer mark up recommendations” (if not I will work with editorial office to get this to you).

Further, the overall flow of the paper could be substantially improved for the reader. Many parts of the Results section of the manuscript are actually descriptions of methods (Figure 1 for example). Some of the text in the results section is also redundant with text in methods.

Minor concerns:

1. The authors’ method of quantifying regrowth past the cut addresses correct guidance during regrowth but falls short of assessing functional regrowth as claimed by the authors.

2. Do microglia migrate to the ES site in non-axotomized animals?

3. Figures 3 and 4 should be merged.

4. Figure 5 and Figure 6 should be merged to reduce redundancy.

5. The choice of statistical tests in specific experiments is not well explained. For example, why is a one-tailed t-test selected when comparing regenerative growth when a two-tailed test is selected for other experiments?
